# Supplementary material for: Structural basis for autoinhibition by the dephosphorylated regulatory domain of Ycf1
Source: Nat Commun. 2024 Mar 16;15:2389. doi: 10.1038/s41467-024-46722-w (PMC10944535; doi:10.1038/s41467-024-46722-w)
Supplement: Supplementary file 1 — Supplementary Info File #1 [file 41467_2024_46722_MOESM1_ESM.pdf]

Supplementary Information

Supplementary Tables

Supplementary Table 1. List of primers used in the study.

FP: Forward primer, RP: Reverse primer

Primer name	5'-3' sequence
E1435Q-FP	CTTGGTTTTGGATCAAGCTACAGCTGCAG
E1435Q-RP	CTGCAGCTGTAGCTTGATCCAAAACCAAG
S869A-FP	GAATTTGGTGACGCTTCAGAATCTTCAG
S869A-RP	CTGAAGATTCTGAAGCGTCACCAAATTC
S878A-FP	CAGTTAGAGAATCTGCAATTCCAGTTGAGGG
S878A-RP	CCCTCAACTGGAATTGCAGATTCTCTAACTG
S903A-FP	GTAACTCTGATGCTATCGCATTGAGAAGAGC
S903A-RP	GCTCTTCTCAATGCGATAGCATCAGAGTTAC
S908A-FP	CATTGAGAAGAGCTGCAGATGCAACTTTGGG
S908A-RP	CCCAAAGTTGCATCTGCAGCTCTTCTCAATG
T911A-FP	GCTTCAGATGCAGCTTTGGGTTCTATCG
T911A-RP	CGATAGAACCCAAAGCTGCATCTGAAGC
S914A-FP	GATGCAACTTTGGGTGCTATCGATTTCGGTGACG
S914A-RP	CGTCACCGAAATCGATAGCACCCAAAGTTGCATC

Supplementary Table2. Cryo-EM data collection, refinement, and validation statistics

	Ycf1-dephos (EMDB-40451) (PDB 8SG4)
**Data collection and processing**	
Magnification	81,000
Voltage (kV)	300
Electron exposure (e–/Å^2)	~52
Defocus range (μm)	-0.6 to -1.9
Pixel size (Å)	1.0694
Symmetry imposed	C1
Initial particle images (no.)	5,424,196
Final particle images (no.)	73,611
Map resolution (Å)	3.11
FSC threshold	0.143
Map resolution range (Å)	2.83-7.08
**Refinement**	
Initial model used (PDB code)	7M69 and AF-P39109-F1
Model resolution (Å)	3.46
FSC threshold	0.5
Map sharpening *B* factor (Å^2)	-85.5
Model composition	
Non-hydrogen atoms	11,715
Protein residues	1466
Ligands	0
B factors (Å^2)	
Protein	85.10
Ligand	-
R.m.s. deviations	
Bond lengths (Å)	0.005
Bond angles (°)	1.006
Validation	
MolProbity score	0.74
Clashscore	0.76
Poor rotamers (%)	0
Ramachandran plot	
Favored (%)	98.09
Allowed (%)	1.91
Disallowed (%)	0

Supplementary Figures
Supplementary Figure 1

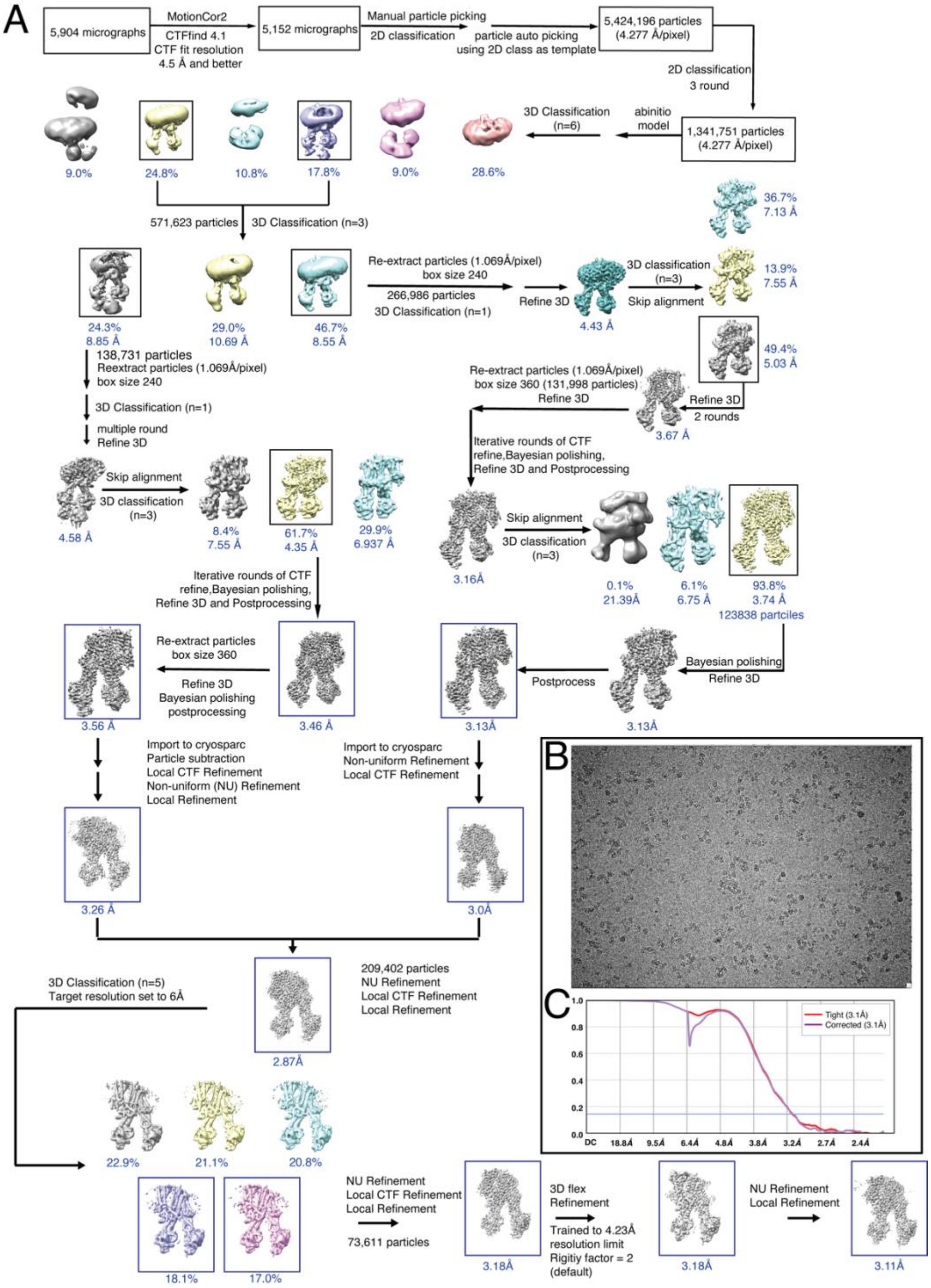

Figure S1: Cryo-EM data processing workflow of dephosphorylated Ycf1. A. Data processing workflow for dephosphorylated *S. Cerevisiae* Ycf1-E1435Q. **B**. Representative cryo-EM micrograph of dephosphorylated Ycf1. **C.** Fourier shell correlation curve with resolution cutoff of 0.143 for the final Cryosparc map used for model building and Phenix refinement.

Supplementary Figure 2

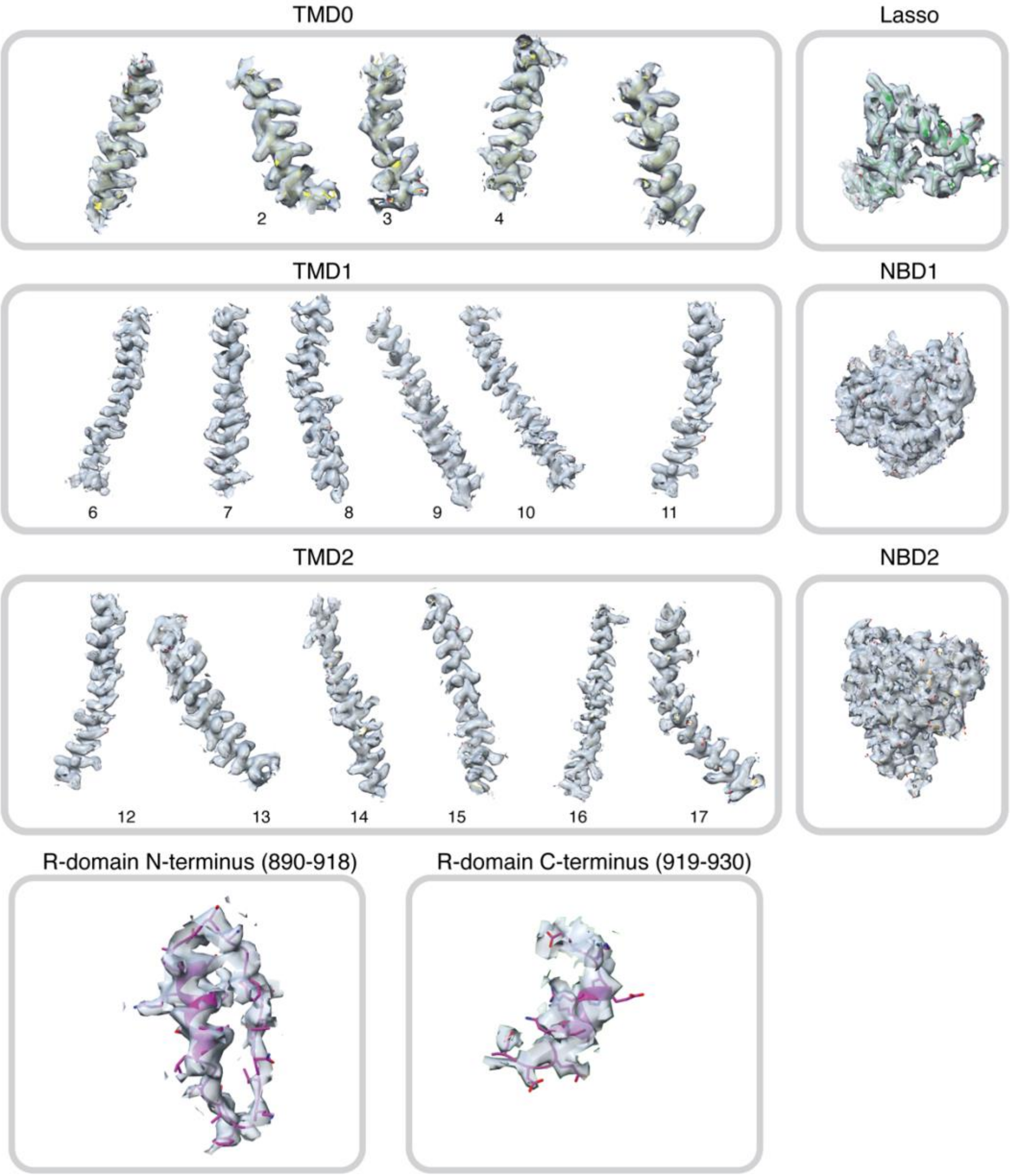

Figure S2: Cryo-EM densities for secondary structure elements and domains of dephosphorylated Ycf1. Cryo-EM density corresponding to secondary structural elements and domains observed in the dephosphorylated Ycf1- E1435Q structure. R-domain density is shown for the N and C-termini. The cryo-EM density in all images is contoured to the same level (4.5 RMSD). The color scheme is identical to main text Figure 1.

Supplementary Figure 3

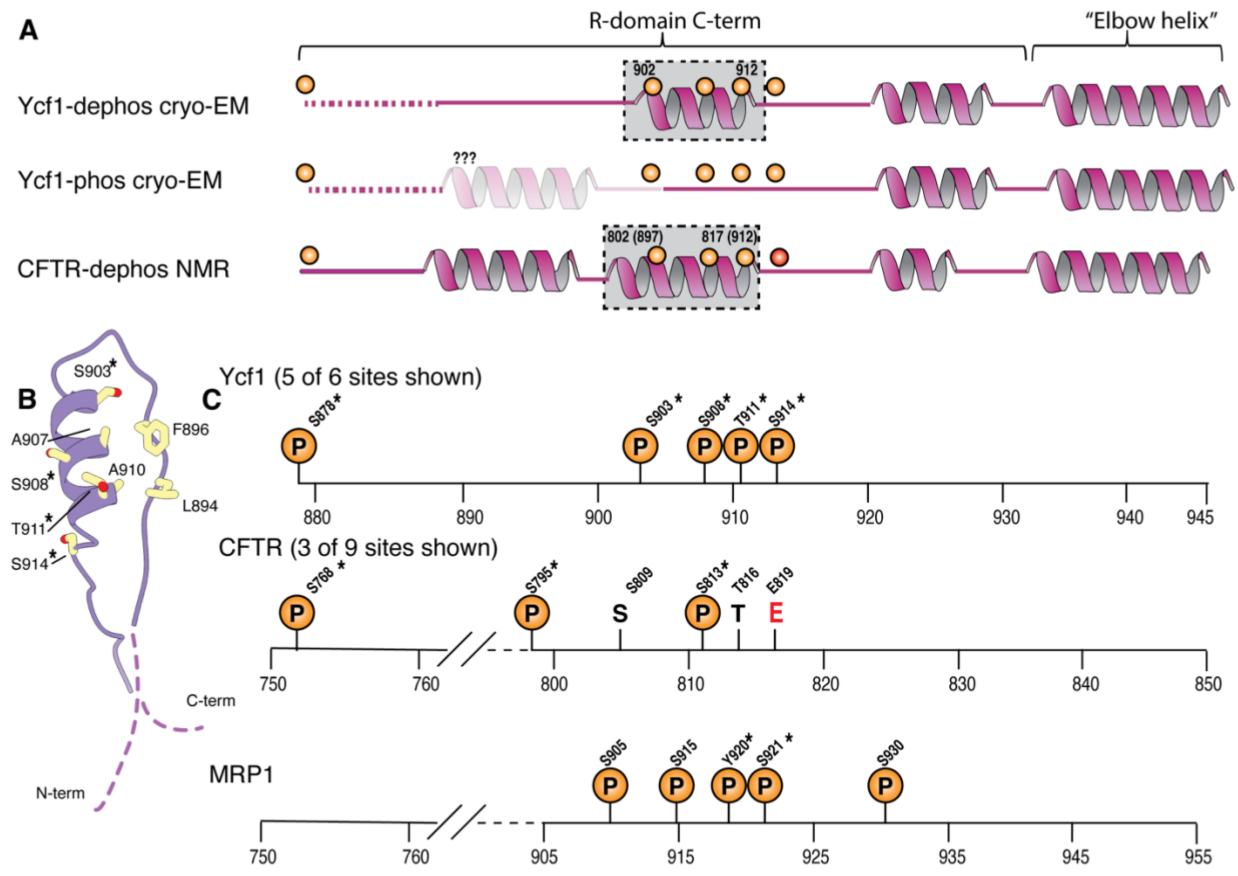

Figure S3: Comparative analysis of secondary structural features of the R-domain. A. Topology of secondary structure in the R-domain C-terminus of Ycf1 and CFTR from cryo-EM structures(this work and our previous phosphorylated Ycf1 structure[1]) or NMR[2], with corresponding phosphorylation sites highlighted with an orange circle. Red circles denote the presence of a glutamate residue where a phosphorylation site exists in the other proteins. The secondary structure shown represents the structurally conserved regions observed in the respective cryo-EM maps, whereas the transparent helix in Ycf1-phos denotes a region for which cryo-EM density was weak and a model could not be built. **B.** Closeup of the Ycf1 R-domain hairpin loop architecture and relative arrangement of phosphorylation sites. Hydrophobic residues forming key interactions are shown as sticks. **C.** Position of phosphorylation sites, conserved residues, and negatively charged amino acids in the R-domain of Ycf1 (top), CFTR (middle), and MRP1 (bottom). Asterix (*) denotes sites confirmed to be phosphorylated.

Supplementary Figure 4

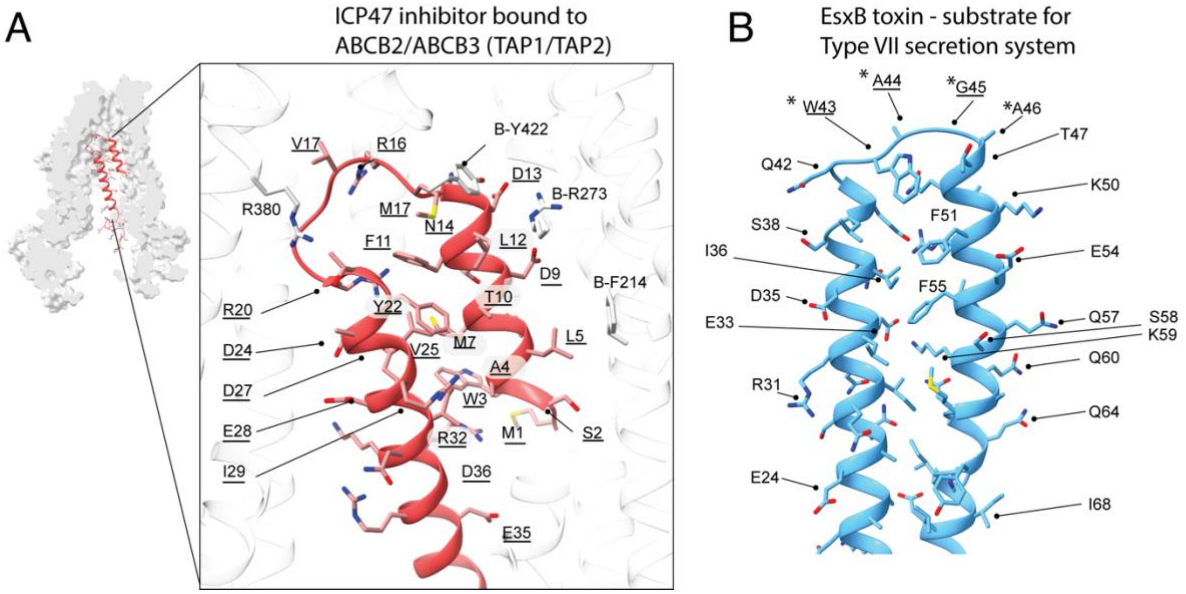

Figure S4: R-domain-like architecture of peptide ligands in an ABC transporter or transporter with ABC-like ATPase domain. A. Specific interactions between the herpes simplex virus IC47 peptide inhibitor (red cartoon) in a closed hairpin loop formation bound to TAP1/TAP2 (grey cartoon; PDB ID:5U1D[3]). Underlined residues denote those on the IC47 peptide itself. **B.** Alphafold2 structure of the type VII secretion system substrate peptide EsxB from *Bacillus antracis*. The symbol (*) denotes residues of the WxG motif.

Supplementary Figure 5

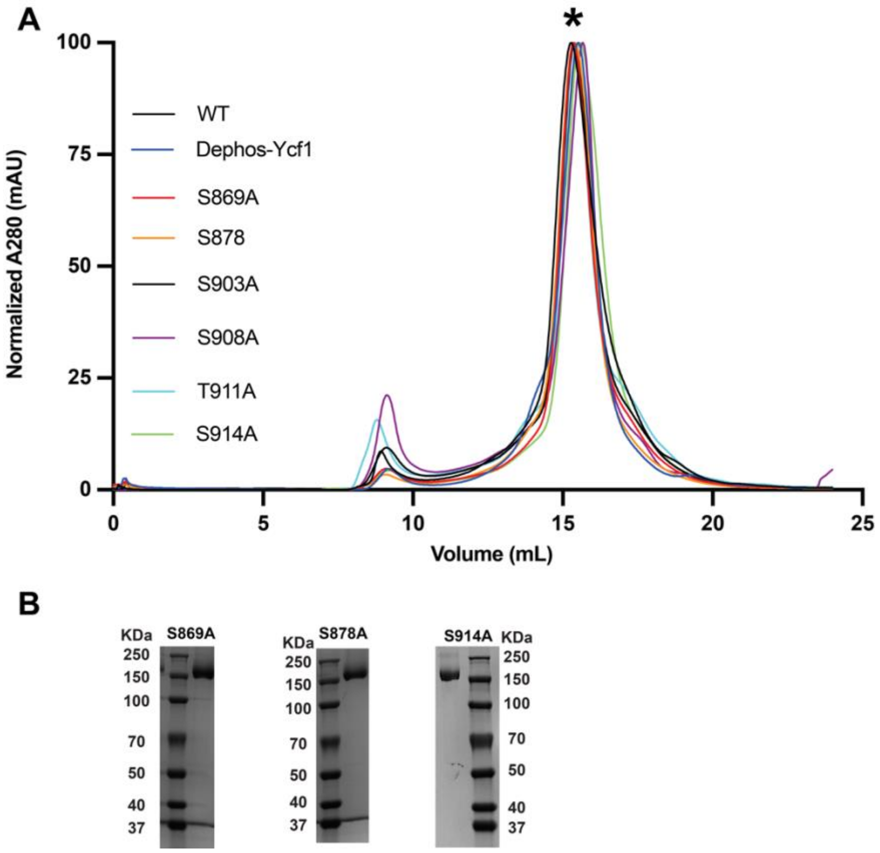

Figure S5: Size exclusion purification profile of Ycf1 and mutant variants. A. Chromatograms are representative of mutants of Ycf1 purified using a Superose 6 Increase 10/300 GL Column (Cytiva). **B.** SDS gel of Ycf1 mutants (S869A, S878A and S914A).

Supplemental References

1. Khandelwal, N. K. *et al.* The structural basis for regulation of the glutathione transporter Ycf1 by regulatory domain phosphorylation. *Nat Commun* **13**, 1278 (2022).

2. Baker, J. M. R. *et al.* CFTR regulatory region interacts with NBD1 predominantly via multiple transient helices. *Nature Structural & Molecular Biology* **14**, 738–745 (2007).

3. Oldham, M. L. *et al.* A mechanism of viral immune evasion revealed by cryo-EM analysis of the TAP transporter. *Nature* **529**, 537–540 (2016).
