# Supplementary material for: Structural basis for autoinhibition by the dephosphorylated regulatory domain of Ycf1
Source: Nat Commun. 2024 Mar 16;15:2389. doi: 10.1038/s41467-024-46722-w (PMC10944535; doi:10.1038/s41467-024-46722-w)
Supplement: Supplementary file 4 — Reporting Summary [file 41467_2024_46722_MOESM4_ESM.pdf]

nature portfolio

Corresponding author(s): Thomas Tomasiak

Last updated by author(s): Feb 14, 2024

Reporting Summary

Nature Portfolio wishes to improve the reproducibility of the work that we publish. This form provides structure for consistency and transparency in reporting. For further information on Nature Portfolio policies, see our Editorial Policies and the Editorial Policy Checklist.

Statistics

For all statistical analyses, confirm that the following items are present in the figure legend, table legend, main text, or Methods section.

n/a	Confirmed	
☐	☒	The exact sample size (n) for each experimental group/condition, given as a discrete number and unit of measurement
☐	☒	A statement on whether measurements were taken from distinct samples or whether the same sample was measured repeatedly
☒	☐	The statistical test(s) used AND whether they are one- or two-sided<br>*Only common tests should be described solely by name; describe more complex techniques in the Methods section.*
☒	☐	A description of all covariates tested
☒	☐	A description of any assumptions or corrections, such as tests of normality and adjustment for multiple comparisons
☐	☒	A full description of the statistical parameters including central tendency (e.g. means) or other basic estimates (e.g. regression coefficient) AND variation (e.g. standard deviation) or associated estimates of uncertainty (e.g. confidence intervals)
☒	☐	For null hypothesis testing, the test statistic (e.g. F, t, r) with confidence intervals, effect sizes, degrees of freedom and P value noted<br>*Give P values as exact values whenever suitable.*
☒	☐	For Bayesian analysis, information on the choice of priors and Markov chain Monte Carlo settings
☒	☐	For hierarchical and complex designs, identification of the appropriate level for tests and full reporting of outcomes
☒	☐	Estimates of effect sizes (e.g. Cohen's d, Pearson's r), indicating how they were calculated

Our web collection on statistics for biologists contains articles on many of the points above.

Software and code

Policy information about availability of computer code

Data collection	Cryo-EM data were collected by staff at the NIH Pacific Northwest Cryo-EM Center (PNCC) on a Thermo Fisher Titan Krios with SerialEM 3.9.0 beta.
Data analysis	RELION 4.0, MotionCor2, CTFFIND4.2, SIDESPLITTER, COOT 0.9, ,Phenix (versions 1.19, 1.19.1-4122-000, 1.20.1-4487-000, phenix-1.20.1-4487), UCSF-ChimeraX (versions 1.1,1.14,1.3,1.4, and 1.5) MolProbity, AlphaFold2, Graphpad prism 9, Isolde (versions 0.93,1.1,1.3, 1.4, and 1.5), and Coot (versions 0.9,0.9-pre EL, 0.9.8.1 , 0.9.2-pre EL), Cryosparc (versions 3.3.1 +220315 and 4.2.1), EMReady (v1.0), 3v 1.2, MapQ

For manuscripts utilizing custom algorithms or software that are central to the research but not yet described in published literature, software must be made available to editors and reviewers. We strongly encourage code deposition in a community repository (e.g. GitHub). See the Nature Portfolio guidelines for submitting code & software for further information.

Data

Policy information about availability of data

All manuscripts must include a data availability statement. This statement should provide the following information, where applicable:

- Accession codes, unique identifiers, or web links for publicly available datasets
- A description of any restrictions on data availability
- For clinical datasets or third party data, please ensure that the statement adheres to our policy

All data are available in the main text or the supplementary materials. Structure presented here is available for download from the Protein Data Bank (PDB codes:

(8SG4) and EM data from the EMDB (EMD-40451).

Research involving human participants, their data, or biological material

Policy information about studies with human participants or human data. See also policy information about sex, gender (identity/presentation), and sexual orientation and race, ethnicity and racism.

Reporting on sex and gender	N/A
Reporting on race, ethnicity, or other socially relevant groupings	N/A
Population characteristics	N/A
Recruitment	N/A
Ethics oversight	N/A

Note that full information on the approval of the study protocol must also be provided in the manuscript.

Field-specific reporting

Please select the one below that is the best fit for your research. If you are not sure, read the appropriate sections before making your selection.

[x] Life sciences [ ] Behavioural & social sciences [ ] Ecological, evolutionary & environmental sciences

For a reference copy of the document with all sections, see nature.com/documents/nr-reporting-summary-flat.pdf

Life sciences study design

All studies must disclose on these points even when the disclosure is negative.

Sample size	Sample size calculations were not performed. Samples sizes were chosen based off of standards from review of literature in the field. All ATPase assays were performed as technical quadruplicates .
Data exclusions	All raw cryo-EM data were processed through the Relion4.0 and Cryosparc pipelines. Micrographs were excluded based on poor resolution estimates using CTFFIND4.2 which often correlated with heavy ice contamination. Particle selection was performed following 2D and 3D classification in Relion and judging by predicted resolution and visual inspection of classes.
Replication	All biochemical experiments were performed at least in triplicate and were performed multiple times. All attempts at replication were successful.
Randomization	Particles were randomized into two half sets following the standard automated Relion4.0 protocol. For biochemical and cellular assays, randomization was not performed. Covariance was controlled by grouping all mutants together and performing analysis at one time.
Blinding	Researchers were not blinded to group allocation. All internal assignment to different classes were performed internally by the reconstruction software (Relion4.0) and particles were assigned to different independent half-maps automatically. Blinding for cryo-EM analysis was not feasible since manual inspection of maps is a necessary aspect of quality determination. For biochemical and cellular studies, no blinding was used owing to the protocols used, which follow the standard in the field, and the necessity to know the sample in order to properly carry out the investigations.

Reporting for specific materials, systems and methods

We require information from authors about some types of materials, experimental systems and methods used in many studies. Here, indicate whether each material, system or method listed is relevant to your study. If you are not sure if a list item applies to your research, read the appropriate section before selecting a response.

Materials & experimental systems

n/a	Involved in the study
☒	☐ Antibodies
☐	☒ Eukaryotic cell lines
☒	☐ Palaeontology and archaeology
☒	☐ Animals and other organisms
☒	☐ Clinical data
☒	☐ Dual use research of concern
☒	☐ Plants

Methods

n/a	Involved in the study
☒	☐ ChIP-seq
☒	☐ Flow cytometry
☒	☐ MRI-based neuroimaging

Eukaryotic cell lines

Policy information about cell lines and Sex and Gender in Research

Cell line source(s)	The DSY-5 yeast strain was obtained by request from the laboratory of Robert Stroud at the University of California - San Francisco.
Authentication	Cell lines are identified by growth on synthetic yeast Histidine dropout media.
Mycoplasma contamination	Yeast cells were not test for contamination as this is typically not a common source of contamination in yeast cells.
Commonly misidentified lines (See ICLAC register)	*Name any commonly misidentified cell lines used in the study and provide a rationale for their use.*
